# Impaired vascular function in gestational diabetes: evidence for metabolic memory, endothelial dysfunction and altered smooth muscle cell biology

**DOI:** 10.3389/fphar.2026.1907433

**Published:** 2026-09-11

**Authors:** Yaw Asare-Amankwah, Marc Bailey, Anne M. Graham, Andrew Tedder, Kirsten Riches-Suman

**Affiliations:** 1 Institute of Health and Social Care, University of Bradford, Bradford, United Kingdom; 2 Leeds Institute of Cardiovascular and Metabolic Medicine, University of Leeds, Leeds, United Kingdom; 3 John Innes Centre, Norwich Science Park, Norwich, United Kingdom

**Keywords:** cardiovascular disease, endothelial cell, gestational diabetes, inflammation, placenta, smooth muscle cell, umbilical cord

## Abstract

Gestational diabetes mellitus (GDM) is a common, inflammatory complication during pregnancy. Women who have GDM are more likely to develop metabolic or cardiovascular disease in the years post-delivery, as are their children. The umbilical cord and placenta provide a unique interface between maternal and foetal circulation and are richly vascularised. However, the molecular and cellular mechanisms that drive metabolic memory and cardiovascular dysfunction in GDM are incompletely understood. This article highlights the clinical presentation of cardiovascular disease post-GDM from mother and child. We investigate histological changes in the vasculature of the umbilical cord and the placenta, and the mechanistic changes in vascular smooth muscle cell and endothelial cell behaviour that may underpin clinical cardiovascular presentations. Finally we suggest areas for future research to increase mechanistic knowledge in this area, which may lead to new personalised medicine approaches for early intervention after delivery to minimise the long-term detrimental effect of GDM on cardiovascular health.

## 1 Introduction

Gestational diabetes (GDM) is a transient condition characterised by abnormal glucose tolerance during pregnancy. It usually presents in the second or third trimesters and resolves after delivery, however it is a leading cause of several complications during pregnancy such as pre-eclampsia and preterm birth. Although transient, both mother and offspring from these pregnancies are much more likely to develop long-term metabolic and cardiovascular dysfunction (Nakshine and Jogdand, 2023) and the mechanisms that lead to this are not fully understood.

GDM presents in 14% of pregnancies globally (Wang H. et al., 2022). Prevalence is associated with numerous risk factors including high body mass index, age at pregnancy, prior GDM pregnancies, familial history, ethnicity, and low socioeconomic background (Wang X. et al., 2022; Amiri et al., 2021; Sweeting et al., 2022). During pregnancy, there is increased maternal insulin demand due to increased insulin resistance, to assist nutrient transport to the growing foetus (Hay, 2006). This is facilitated by an increase in hormones including leptin, cortisol, progesterone, oestrogen and placental growth hormone (Handwerger and Freemark, 2000; Newbern and Freemark, 2011). A decrease in insulin sensitivity of approximately 60% is observed in both women with normal glucose tolerance and GDM. To compensate for this, a physiological adaptation in the pancreatic and β-cells in the islet of Langerhans enhances insulin secretion. However, a failure in β-cell and overall pancreatic adaptation further exacerbated by factors such as obesity can contribute to insufficient insulin levels, leading to GDM (Catalano, 2014). Indeed, clinical observations have shown that β-cell adaptation in Type 1 diabetes mellitus (T1DM) during pregnancy restored endogenous insulin production during pregnancy (Espes et al., 2022). This highlights the importance of β-cell adaptation in maintaining glucose homeostasis during pregnancy with its dysregulation having serious consequences for proper glucose regulation as well as maternal and foetal health.

In addition to pancreatic adaptations to facilitate glycaemic control, the vasculature of the mother adapts to accommodate the growing foetus. This process typically involves hormonal alterations which result in increased blood volume, heart rate, systemic vascular resistance, decreased mean arterial pressure (MAP) and increased cardiac output to optimise oxygen and nutrient delivery to the foetus (Meah et al., 2016; Moore et al., 2024; Robson et al., 1989; Sanghavi and Rutherford, 2014). For instance, to promote unrestricted blood flow and reduce vascular resistance, vasodilation mediated by nitric oxide (NO), relaxin, oestrogen and progesterone begins as early as the fifth week of gestation (Chapman et al., 1998; Esposito et al., 2025; Leo et al., 2017). These hormonal changes ensure an increased plasma volume as compared to pre-pregnancy. Cardiac output also peaks in the early third trimester and gradually reduces towards the end of term as a result of foetal growth. This non-linear behaviour is however temporary, and cardiac output reduces considerably after delivery due to reduced demand on maternal vasculature. Despite this increase in cardiac output, blood pressure decreases as a result of the decreased vascular resistance (Mulder et al., 2022). Haemodynamic changes in GDM-related pregnancies present a unique challenge to both the mother and foetus. There is evidence of increased aortic stiffness in women who previously had GDM compared to women without, which is an indicator of adverse diabetes complications (Lekva et al., 2015). Additionally, there is higher vascular resistance associated with GDM compared to non-diabetes pregnancies, which negatively influences blood flow and pressure (Mulder et al., 2022). These changes suggest maladaptation at the cellular level which remain to be fully elucidated.

The comorbidities associated with GDM include obstetrical, maternal and foetal complications as evidenced in multiple studies. The co-existence of GDM and hypertensive disorders of pregnancy represents a high-risk obstetric phenotype compared to each condition alone (Lin et al., 2024). This has implications for foetal outcomes with increased odds of abnormal foetal growth and preterm birth. Similarly, poor clinical outcomes are often evident in pregnancies complicated by both obesity and GDM. Obesity has been shown to amplify the adverse neonatal effects of GDM leading to increased risk of macrosomia, larger for gestational age (LGA), preterm birth and shoulder dystocia [reviewed in Weir et al. (2024)]. These findings suggest that the coexistence of these disorders appear to confer substantially greater risk to both the mother and the foetus.

As well as the increased morbidity associated with GDM, pregnancies with the condition are estimated to increase the healthcare costs for pre- and post-delivery care (Dinh et al., 2024) and may cost the UK National Health Service close to £150,000,000 per year (Hex et al., 2024). Hence, as well as the personal impact of GDM, it has a significant impact on social economics and healthcare costs. The first line treatment for patients with GDM is lifestyle modifications to try to normalise glycaemic control. However, if this is unsuccessful, pharmacological interventions can be prescribed [extensively recently reviewed in Albairmani et al. (2025) and Gautam et al. (2025)]. The most common treatments are insulin, metformin and glibenclamide (glyburide). Insulin supplementation is commonplace for other forms of diabetes (T1DM and type 2 diabetes; T2DM) and is also considered safe in GDM as insulin does not cross the placental barrier. As well as restoring glucose control, studies have shown that insulin treatment can reduce the likelihood of pregnant women developing pre-eclampsia (Yang and Wu, 2022) and it has systemic anti-inflammatory effects (Sun et al., 2014).

Unlike insulin, both metformin and glibenclamide can cross the placental barrier and enter foetal circulation. Metformin is a biguanide that increases glucose absorption by the gut, thereby reducing the amount of glucose that remains in the circulation (Du et al., 2022). The impact of metformin on developing pre-eclampsia appears to be on a par with insulin treatment (Yang and Wu, 2022). Limited studies have provided long-term follow up on the offspring from metformin-treated pregnancies, but there is a suggestion that *in utero* exposure to metformin is associated with increased weight in childhood, and possible changes in steroid hormone management in male offspring (Toft and Økland, 2024). Glibenclamide is a sulphonylurea. It increases the production of insulin and acts as an insulin-sensitiser in peripheral tissues. This helps to maintain blood glucose within a normal range (Lv et al., 2020). It is potentially less effective than insulin in preventing pre-eclampsia (Yang and Wu, 2022). Glibenclamide has been associated with adverse pregnancy outcomes including LGA deliveries and neonatal hypoglycamia (Bodier et al., 2025; Camelo Castillo et al., 2015).

This review will highlight current knowledge around the cardiovascular complications of GDM for both mother and child. It will examine how GDM impacts on the umbilical and placental vasculature and identify cellular and molecular mechanisms that underpin this. It will also demonstrate the significant gaps in current understanding that need to be addressed to ensure pregnant women and their children receive the best care possible to counter cardiometabolic consequences of GDM pregnancies.

## 2 Cardiovascular changes for mothers with GDM

Whilst GDM usually resolves after delivery, it has a lasting impact on the cardiometabolic system of the mother. Women who have had GDM have a 2x greater risk of developing cardiovascular disease than women who had normoglycaemic pregnancies (Kramer et al., 2019). Pregnancy acts as a stress test for the heart, with increased cardiac output, left ventricular (LV) mass and LV end-diastolic volume observable even in the earliest stages [reviewed in Sanghavi and Rutherford (2014)]. These essential adaptive responses to pregnancy can become maladaptive in GDM.

In women with GDM, echocardiographic studies have demonstrated subclinical LV systolic and diastolic dysfunction, with reductions in stroke volume and cardiac output in some cohorts (Sonaglioni et al., 2024). In contrast, other studies focussing on women with GDM and pre-existing diabetes, have reported higher median cardiac output and heart rate compared with women without GDM or pre-existing diabetes (Szczepkowska et al., 2024), suggesting a constantly changing haemodynamic profile in this subgroup. Altogether, these findings indicate that cardiac involvement in GDM is heterogeneous, ranging from subtle ventricular dysfunction to a hyperdynamic circulatory state. These have negative cardiovascular outcomes such as myocardial infarction, ischaemic stroke, peripheral artery disease, heart failure and hypertension in women with pre-existing diabetes or previous GDM (Yu et al., 2022).

At the vascular level, circulation via arterial conduits is heavily altered mainly due to arterial stiffness as well as subsequent development of pre-eclampsia (Robb et al., 2009; Mansukhani et al., 2024). Two commonly used methods for assessing vascular stiffness are pulse-wave velocity (PWV) and augmentation index (AIx). PWV measures arterial blood flow velocity and is calculated by occluding two arterial vessels (e.g., femoral, carotid) and measuring the time it takes for the pulse wave to travel between the two. AIx is simpler in that only one vessel is occluded and measures the augmentation of central aortic pressure by a reflected pulse wave (Russell et al., 2024). There is, however, conflicting evidence on indicators of arterial stiffness during GDM.

One study demonstrated a significant increase in carotid-femoral PWV in GDM mothers compared to control pregnancies (Mansukhani et al., 2023) (Table 1). However, other studies have found only modest increases in carotid-femoral (Salmi et al., 2012; Savvidou et al., 2010) and carotid-radial PWV (Garg et al., 2017) that were not significant. Aortic PWV is reportedly comparable between GDM mothers and non-diabetes controls (Anness et al., 2023; Bulzico et al., 2012). Very recently, PWV was found to be decreased in GDM mothers specifically during the third trimester (Dugandžić Šimić et al., 2026).

**TABLE 1:** Pulse-wave velocity measurements in GDM. Summary table of published data on the impact of GDM on pulse wave velocity (PWV), stratified for trimester.

Trimester	% Change	n number	Sig.	Measurement	References
First	↑ 11.2%	20 GDM 20 controls	ns	Carotid-radial	Garg et al. (2017)
Second	↑ 6.0%	20 GDM 20 controls	ns	Carotid-radial	Garg et al. (2017)
Second	↑ ∼1.6%	127 GDM 155 controls	ns	Aortic	Anness et al. (2023)
Third	↑ 3.6%	218 GDM 1800 controls	*	Carotid-femoral	Mansukhani et al. (2023)
Third	↑ 3.9%	22 GDM 31 controls	ns	Carotid-femoral	Salmi et al. (2012)
Third	↑ 11.1%	34 GDM 34 controls	ns	Carotid-femoral	Savvidou et al. (2010)
Third	↓ 6.4%	50 GDM 50 controls	*	Carotid-femoral	Dugandžić Šimić et al. (2026)
Third	↑ 9.3%	20 GDM 20 controls	ns	Carotid-radial	Garg et al. (2017)
Third	↑ 6.6%	34 GDM 34 controls	ns	Carotid-radial	Savvidou et al. (2010)
Third	↑ ∼1.5%	127 GDM 155 controls	ns	Aortic	Anness et al. (2023)
Not stated	↑ 1.4%	24 GDM 27 controls	ns	Aortic	Bulzico et al. (2012)

Differences observed across studies may be influenced by variations in patient characteristics (age, ethnicity, body mass index), study design, methodology for assessing PWV and lifestyle/pharmacological interventions.

*p < 0.05, ns = not significant.

GDM-related changes in AIx appear slightly more consistent, but there is still a lack of consensus (Table 2). Significant increases in AIx have been reported that are unaffected by interventions (Savvidou et al., 2010; Garg et al., 2017; Anness et al., 2023). More subtle or insignificant variability has been reported by others (Mansukhani et al., 2023; Salmi et al., 2012; Dugandžić Šimić et al., 2026).

**TABLE 2:** Augmentation index measurements in GDM. Summary table of published data on the impact of GDM on augmentation index (AIx), stratified for trimester.

Trimester	% Change	n number	Sig.	Measurement	References
First	↑ 237%	20 GDM 20 controls	*	Central AIx	Garg et al. (2017)
Second	↑ 600%	20 GDM 20 controls	*	Central AIx	Garg et al. (2017)
Second	↑	127 GDM 155 controls	***	Aortic AIx	Anness et al. (2023)
Second	↑	127 GDM 155 controls	***	Brachial AIx	Anness et al. (2023)
Third	↑	127 GDM 155 controls	***	Aortic AIx	Anness et al. (2023)
Third	↑	127 GDM 155 controls	***	Brachial AIx	Anness et al. (2023)
Third	↑ 1.4%	50 GDM 50 controls	ns	Brachial AIx	Dugandžić Šimić et al. (2026)
Third	↑ 185%	20 GDM 20 controls	*	Central AIx	Garg et al. (2017)
Third	↑ 1871%	34 GDM 34 controls	***	Central AIx	Savvidou et al. (2010)
Third	↑ 3.7%	22 GDM 31 controls	ns	Central AIx	Salmi et al. (2012)
Third	↓ 12.1%	218 GDM 1800 controls	ns	AIx delta	Mansukhani et al. (2023)

Differences observed across studies may be influenced by variations in patient characteristics (age, ethnicity, body mass index), study design, methodology for assessing AIx and lifestyle/pharmacological interventions.

***p < 0.001, *p < 0.05, ns = not significant.

It remains to be seen whether vascular stiffness can be conclusively observed in GDM. It may be that any changes are so subtle that current studies are underpowered which might limit clinical usefulness. Variations in vascular stiffness caused by age, ethnicity, and different interventions such as diet, insulin therapy or metformin may mask or confound direct effects of GDM, which requires further study if it is to be a valuable prediction of postpartum cardiovascular risk for the mother.

Whilst vascular stiffness may be variable, what is more certain is the increased risk of cardiovascular disease development, particularly atherosclerosis and vascular calcification, in mothers who have experienced GDM (Brewster et al., 2013; Gunderson et al., 2021; Parikh et al., 2021). Even when mothers do not progress to T2DM, the existence of prior GDM predisposes to cardiovascular disease indicating that even transient, months-long perturbations in glucose homeostasis can have a lasting impact on vascular health (Sweeting et al., 2022; Brewster et al., 2013; Wilson et al., 2022). This is also supported by recent studies where vascular stiffness (AIx) was significantly increased in GDM mothers 2 months after birth (Dugandžić Šimić et al., 2026), suggesting the concept of ‘metabolic memory’.

## 3 Cardiovascular changes for the offspring of GDM pregnancies

In addition to lasting cardiovascular burden on mothers, long-term complications for the offspring are also a major concern [reviewed in Wicklow and Retnakaran (2023)]. Increased blood glucose crosses the placenta and stimulates foetal insulin production, producing accelerated growth in the foetus as the excess insulin acts as a growth factor, resulting in macrosomia [reviewed by Hufnagel et al. (2022)]. The neonate is also susceptible to hypoglycaemia with studies identifying higher incidences especially with glucose control interventions for mothers with GDM (Kole et al., 2020). Offspring from GDM pregnancies have a high risk of presenting congenital heart disease (Wu et al., 2020) with adverse effects on overall cardiovascular development as a result of foetal exposure to high blood glucose levels *in utero* and additional long-term risks such as obesity (Liu et al., 2026).

The Hyperglycaemia and Adverse Pregnancy Outcomes (HAPO) study (Lowe et al., 2019a) revealed a strong connection between maternal hyperglycaemia and insulin resistance and hyperglycaemia in children as they developed. Children are more likely to be overweight or obese, with early indicators of metabolic dysfunction and elevated glycosylated haemoglobin (Lowe et al., 2019a; Lowe et al., 2019b; Scholtens et al., 2019). Clinically, there is evidence of increased intimal-medial thickness from birth and during childhood (Koklu et al., 2007; Yapicioglu et al., 2023) and increased vascular stiffness (Boehme et al., 2022). These changes and complications present a burden during childhood with potential progression into and during adulthood.

**FIGURE 1:**
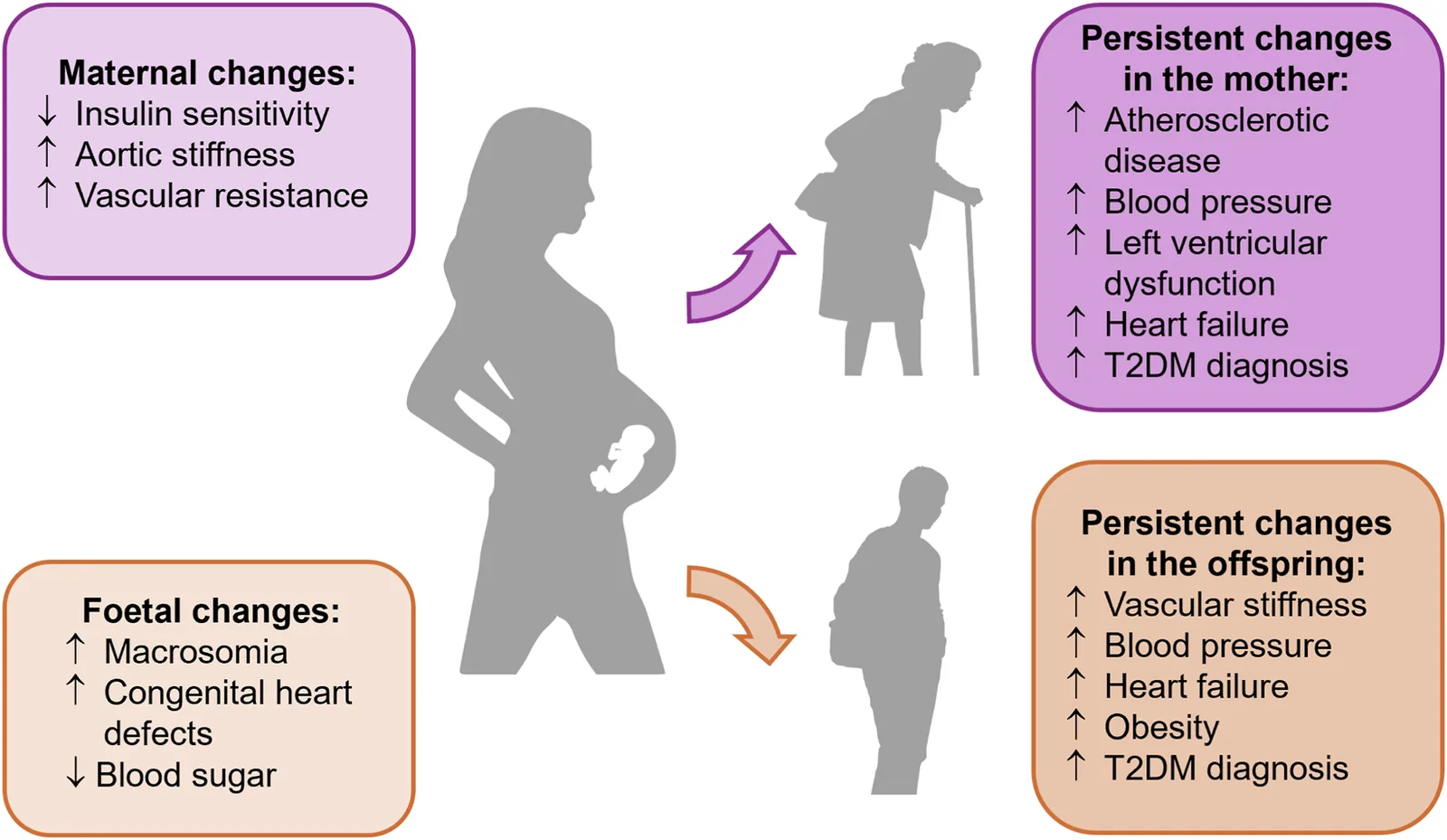
Clinical and subclinical parameters in mothers and offspring resulting from GDM pregnancies. Cardiovascular and metabolic changes are seen in mother and foetus during pregnancy, and in both mother and child following birth.

There are a few studies that have examined cardiovascular outcomes in the offspring of GDM pregnancies once they have reached adulthood. These reveal an increase in the risk of cardiovascular disease including hypertension, myocardial infarction and heart failure in midlife (aged 35–40) (Yu et al., 2022; Guillemette et al., 2020; Yu et al., 2019). Thus, as well as an increased risk of cardiovascular disease for mothers exposed to transient hyperglycaemia, the same is apparent for offspring where exposure to the diabetic milieu during foetal development has a persistent effect. The clinical and sub-clinical outcomes for mother and child both during and after pregnancy are summarised in Figure 1.

## 4 Metabolic memory

Given the prolonged impact of transient hyperglycaemia on both mother and offspring, it is important to consider the concept of metabolic memory. In this context, metabolic memory is a term that describes the long-term physiological and metabolic effects that prenatal environments have on vascular health. This phenomenon is particularly significant in GDM given the persistent metabolic alterations even after maternal blood glucose levels normalise post-pregnancy.

The influence of metabolic memory on cardiovascular health in T2DM is well recognised. Patients with T2DM are predisposed to earlier development of cardiovascular disease and, even when glucose levels are well controlled, this increased risk persists (Eckel et al., 2021). This may in part be due to the persistence of functional differences in vascular cells from patients with T2DM. Smooth muscle cells (SMC) from the saphenous vein of patients with T2DM are known to have persistent changes in cellular proliferation, senescence and inflammatory cytokine release in the absence of glucose manipulation, as well as dysfunctional cytoskeletal fibres that can affect vascular function. These are mediated, at least in part, by persistent elevations in microRNAs miR-143 and miR-145, reduced expression of RhoA, and hyper-activation of the DNA damage response (Faries et al., 2001; Hemmings et al., 2021; Madi et al., 2009; Riches et al., 2014a; Riches et al., 2014b). Endothelial cells (EC) are similarly affected, with persistent changes in proliferation, migration and inflammatory responses in the absence of glucose stimulation. In this case, inhibition of the Akt and endothelial nitric oxide synthase (eNOS) signalling pathways are apparent, with reduced uptake of oxidized low-density lipoproteins (Cutiongco et al., 2018; Roberts et al., 2015).

One of the challenges in T2DM is that, as it develops silently often for many years prior to diagnosis, there is no way of knowing how long the cardiovascular system has been exposed to the diabetic milieu. The literature does not precisely define the duration of exposure to hyperglycaemia (and other metabolic factors) necessary to induce metabolic memory with some evidence suggesting that even short-term exposures can trigger lasting cellular memory (Wilson-Verdugo et al., 2024). In GDM, the duration of exposure to the diabetic milieu is a maximum of 9 months and appears to be of sufficient duration to inflict persistent harm.

Epigenetic modifications such as DNA methylation, histone modification, and non-coding RNA expression have been shown to be responsible for metabolic memory (Chen and Natarajan, 2022). Analysis of placental tissue and umbilical cord blood has revealed changes in DNA methylation patterns between GDM pregnancies and those without a diagnosis of diabetes. These alterations are linked with metabolic disorders (Awamleh et al., 2021; Lu et al., 2022). Similarly, multiple microRNAs associated with insulin resistance, insulin sensitivity, glucose homeostasis, inflammation and cholesterol biology have been detected in GDM blood samples during pregnancy, and in placental tissue after delivery [reviewed by Elhag and Al Khodor (2023)]. Transcriptomic and subsequent proteomic changes in vascular development and immune signalling have also been recently described (Fakonti et al., 2026).

The evidence for epigenetic modification in GDM, alongside the known propensity for altered function in cardiovascular cell types in T2DM, lay the foundation for understanding metabolic memory and vascular adaptation in GDM, as well as providing insights into potential diagnostic pathways by specifically targeting them as biomarkers. Understanding changes in vascular cells and tissues during GDM is therefore critical for the development of future therapies that may ameliorate the persistent vascular impact of GDM on both mother and child.

### 4.1 Immune-vascular interactions in GDM

In addition to metabolic dysfunction, chronic low-grade inflammation plays a role in the pathogenesis of GDM promoting insulin resistance and impaired foetal development (Xuan Nguyen et al., 2023). This is characterised by altered immune cell activation, and increased circulation of pro-inflammatory cytokines such as TNF-α, IL-1β, and IL-6 are consistently implicated in the pathogenesis of GDM with studies revealing elevated levels in placental tissues driven by hyperglyceamia (Ray et al., 2024; Zgutka et al., 2024). These inflammatory signals could have consequences on the vascular wall as both TNF-α and IL-6 are implicated in impaired endothelial-dependent vasodilation and reduced endothelial function in women with GDM (Mrizak et al., 2013).

In addition to their systemic effects, cytokines released as a result of platelet activation may also promote endothelial activation characterised by increased oxidative stress, reduced NO bioavailability, and enhanced recruitment of circulating leukocytes with emerging evidence showing increased platelet activation and hyperactivity in women with GDM accompanied by impaired NO signalling and endothelial dysfunction (Guglielmini et al., 2025; McElwain et al., 2020). Furthermore, endothelial inflammation may promote SMC phenotypic switching from a contractile to a pro-inflammatory synthetic state characterised by increased proliferation, migration and extracellular matrix production - all processes involved in vascular remodelling (Bennett et al., 2016; Owens et al., 2004).

## 5 Histological changes in the umbilical cord

The umbilical cord is the link between the placenta and the foetus and provides blood flow between the two. The cord is fully formed by the end of the first trimester and is on average 50–60 cm long at delivery. It consists of one umbilical vein and two umbilical arteries embedded within Wharton’s jelly (a mucous connective tissue) and surrounded by single layer of amnion. The single umbilical vein is larger than the arteries and transports oxygenated blood from the placenta to the foetus. Structurally, the umbilical vein has a thickened muscular wall rich in SMC and an internal elastic lamina. By contrast, the umbilical arteries are smaller and lack both internal and external elastic lamina. They coil around the umbilical vein with an average coil length of 5 cm and are responsible for returning oxygen-depleted blood back to the placenta. Adventitial layers are absent, and instead the supporting function of the adventitia is replaced by the Wharton’s jelly (reviewed by Spurway et al., 2012).

Defective vascular structure of the umbilical cord in GDM has been recognised for decades with evidence of extravasation of arterial blood into the Wharton’s jelly (Devi Singh, 1986; Tenaw, 2022). In GDM, umbilical cords present with an increased diameter, and an increased likelihood of having a swollen, oedematous morphology which may be attributed to changes in Wharton’s jelly (Devi Singh, 1986; Ennazhiyil et al., 2025; Kadivar et al., 2020; Weissman and Jakobi, 1997). However, the gross histological changes seen in GDM cords are reportedly much milder than those observed in pregnancies affected by pre-pregnancy diabetes (Karaca et al., 2020).

Studies have shown that vascular coiling in the umbilical cord can be affected by GDM. Noncoiling has been significantly associated with GDM (Ezimokhai et al., 2000; Ezimokhai et al., 2001). Whilst hypercoiling was also previously suggested to be associated with GDM (Ezimokhai et al., 2001), more recent larger studies have shown no such correlation (Kadivar et al., 2020; Najafi et al., 2019; Pooransari et al., 2020). There are also differences in the responses of umbilical arteries and veins to GDM. Arteries are significantly thicker in GDM cords with an increase in both wall thickness and lumen diameter. Whilst thicker walls may be associated with fibrosis, the elastin content is significantly reduced (Ennazhiyil et al., 2025). This suggests alternative mechanisms for thickening, perhaps through phenotypic changes in SMC and hyperplasia. The umbilical vein is more dilated in GDM, however in contrast to the artery, intimal thickness is maintained and it is only the lumen diameter that becomes larger (Devi Singh, 1986; Ennazhiyil et al., 2025). This suggests a loss of vascular tone which can again be due to SMC and EC dysfunction. This is consistent with findings in pregnancy-induced hypertension where there was an observed increase in the wall thickness of the umbilical vein and umbilical arterial wall (Koech et al., 2008).

Given the histological and functional changes seen in the umbilical cord, it is pertinent to evaluate how GDM affects the vascular cells that comprise the umbilical arteries and veins, in order to understand how these morphological changes in tissue may arise.

### 5.1 Molecular changes in vascular cord cells

The integrity of the blood vessel wall is crucial for normal vascular function and when compromised, poses a great challenge. Hyperglycaemia is known to significantly impact vascular cells within the blood vessel and can drive early vascular ageing and vascular injury (Riches-Suman and Hussain, 2022). During healthy pregnancies, endothelial function improves to accommodate the changing haemodynamic demands; this appears compromised in GDM.

The intimal layer lining vessels is made up of EC. These mechano-sense alterations in vascular flow and in response secrete factors that cause contraction or relaxation in SMC to alter vascular tone. Given its role, dysfunction of EC disrupts the continuous flow of blood through the umbilical cord and several pregnancy related disorders including GDM can be linked to EC dysfunction. Human umbilical vein endothelial cells (HUVEC) are very commonly used in culture and have revealed that GDM can influence normal endothelial function by disrupting insulin-mediated vasodilation by the Akt/eNOS pathway (Hengst et al., 2025). Disruption in vasodilation can impact negatively on flow-mediated dilation producing effects such as reduced serum NO levels and increased serum endothelin-1 (ET-1). Together, this would result in vascular contraction and could contribute to poor pregnancy outcomes [reviewed by Bourque et al. (2011)]. However, the effect of GDM on HUVEC NO production is far from clear with evidence that NO synthase activity and expression of endothelial NO synthase is actually increased [reviewed by Sobrevia et al. (2016)]. Functionally, GDM HUVEC consistently present with impaired proliferative and tube-forming capacity (Amrithraj et al., 2017; Sultan et al., 2015). This may be due to a change in the balance of adhesion molecules with GDM-EC expressing higher levels of VE-cadherin, but lower levels of CD44. It may also be due to decreases in apoptotic protein BCL-xL (Amrithraj et al., 2017; Byford et al., 2026). Other signalling pathways such as p44/42 mitogen-activated protein kinase (MAPK) and adenosine have also been reported to be altered in GDM, with an increase in reactive oxygen species (ROS) and impaired mitochondrial function (Sobrevia et al., 2016; Amrithraj et al., 2017; Sultan et al., 2015; Chapple et al., 2015). How these are responsible for HUVEC dysfunction remains to be delineated.

Less is known about human umbilical artery endothelial cells (HUAEC), however they do reportedly present with increased oxidative stress and altered antioxidant systems. This can lead to foetal vascular dysfunction and suggests potential long-term effects on the newborn’s health and development (Carrasco-Wong et al., 2019). Taken together, these findings demonstrate that ECs when compromised can influence the vascular environment through their secretions and the aberrant regulation of essential functional pathways, potentially having negative lasting effects on both the mother and the foetus.

Compared to EC, SMC from the umbilical cord are far less studied. Human umbilical artery SMC (HUASMC) from GDM pregnancies have altered patterns of inter- and intracellular signalling molecules. This includes increased adenosine transport (Aguayo et al., 2001; Aguayo and Sobrevia, 2000), reduced potassium channel expression (both ATP and calcium-dependent; (Li et al., 2022; Li et al., 2018)), and a switch from cAMP to cGMP signalling (Aguayo et al., 2001; Aguayo and Sobrevia, 2000). Functionally, this can contribute to impaired SMC relaxation and increased migration (Ennazhiyil et al., 2025).

Much less is known about human umbilical vein SMC (HUVSMC), although they are reported to be hypermigratory with similarly-reduced K_ATP_ channel expression (Ennazhiyil et al., 2025; Djokic et al., 2019). Combined, this HUASMC and HUVSMC dysfunction can lead to increased vascular stiffness and a higher risk of cardiovascular events in affected individuals however, these may involve different mechanisms due to the delicate yet complex nature of gestation. Nevertheless, prolonged hyperglycaemia has been shown to alter calcium ion (Ca^2+^) signalling in vascular SMC by inhibition of the passive endoplasmic reticulum Ca^2+^ leak (El-Najjar et al., 2017). Given the aforementioned inhibition of calcium-sensitive potassium channels (Li et al., 2022; Li et al., 2018), this combination could conceivably affect contractility and overall vascular health. ROS are also implicated in vascular SMC with elevated levels and accumulation as a result of hyperglycaemia altering proliferation, as well as contractility of SMC [reviewed by Shi et al. (2020)] pertinent to the maladaptation observed in GDM.

**FIGURE 2:**
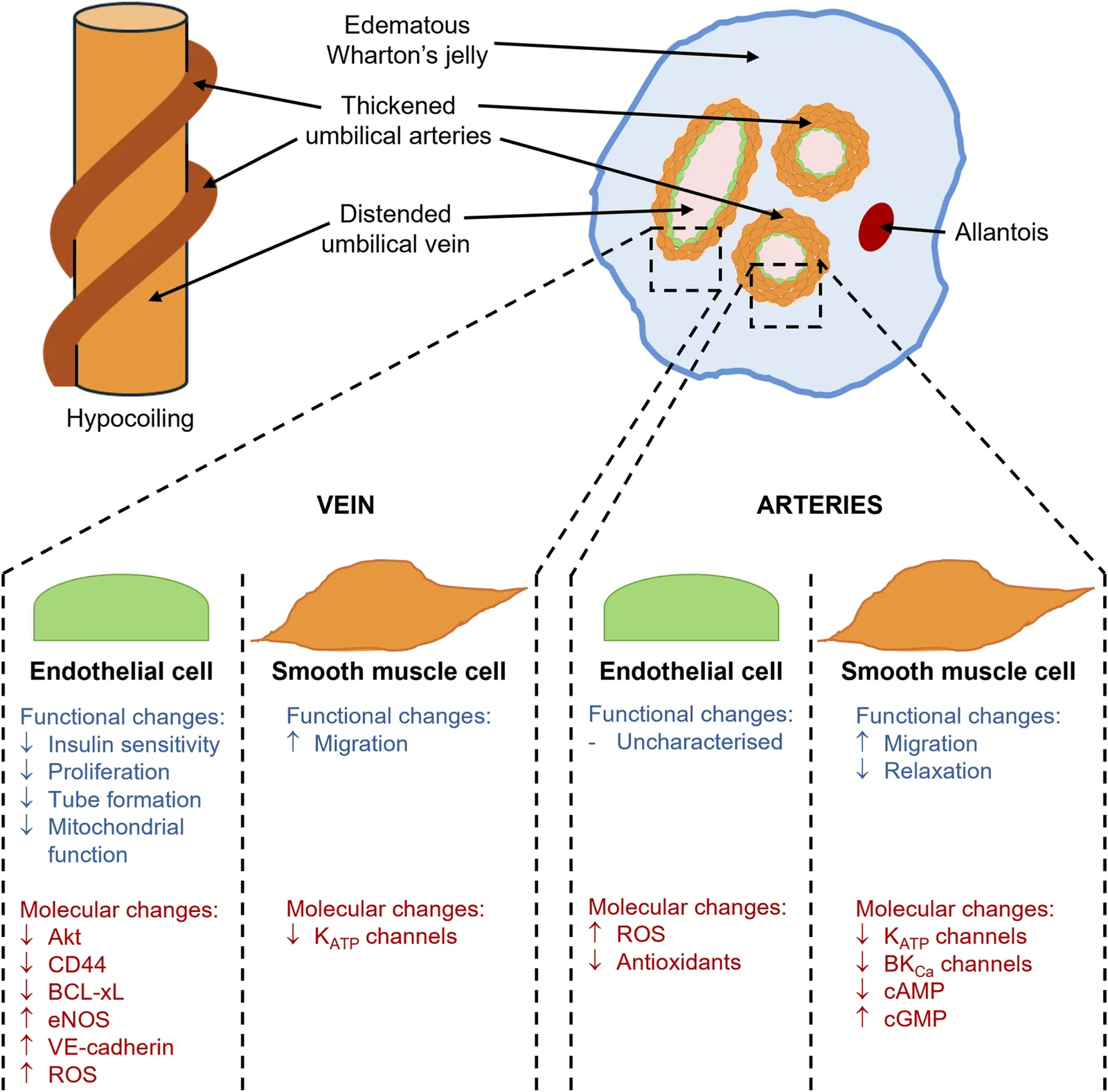
The impact of GDM on the umbilical cord. Histological changes in the umbilical cord such as coiling defects and distension of the umbilical vein are well-recognised. At the cellular level, endothelial cells are affected differently according to source. Increased ROS is common to both, whilst HUVEC specifically have functional issues with reduced proliferation and tube-forming capacity. Research on the impact of GDM on smooth muscle cell phenotype is comparably scarce. Whilst changes in signalling pathways are reported, how this impacts SMC function is more opaque. BK_Ca_, calcium-sensitive potassium channel; cAMP, cyclic adenosine monophosphate; cGMP, cyclic guanine monophosphate; K_ATP_, ATP-sensitive potassium channel; NO, nitric oxide; ROS, reactive oxygen species.

A summary of the histological and cellular changes in the umbilical cord in GDM can be found in Figure 2.

## 6 Histological changes in the placenta

The placenta is a discoid, transient organ that provides the interface between the mother and the developing foetus. It is created from the zygote and belongs genetically to the foetus. At delivery, the human placenta is around 22 cm in diameter and 2.5 cm thick at the centre. Structurally, it has two sides (termed plates) – the chorionic plate faces the foetus and is attached to the umbilical cord, whilst the basal plate embeds within the maternal endometrial wall. These plates are connected through the intervillous space by globular lobules termed villous trees. The stem of these trees originates in the chorionic plate and diverges into ever smaller branches. These are richly vascularised with foetal capillaries. Each of these lobules sit in close proximity to a maternal spiral artery through the basal plate and provide an efficient mechanism of maternal-foetal exchange. Given this structure and its functional importance, the placenta is highly vascularised and at delivery can have a combined blood vessel length of up to 500 km [reviewed by Burton and Fowden (2015)]. The placenta is important for overall pregnancy success and its dysfunction impairs the transfer of vital nutrients thus contributing to pregnancy complications with negative outcomes for both mother and offspring.

The impact of GDM on placental function has revealed conflicting evidence. Some studies have revealed hyperinflammation, oxidative stress, both increased angiogenesis and increased frequency of avascularity in the villous trees, and an increased risk of both maternal and foetal vascular malformation in GDM placentas (Goto et al., 2022; Jarmuzek et al., 2015). Similarly, earlier studies suggested that placental blood vessels were hyperreactive to vasoconstrictors and vasorelaxants independently of the endothelium (Figueroa et al., 1993). Very recently, villous branching has been investigated. Even in well controlled GDM pregnancies, the placentas showed reduced villous branching indexes. Additionally, GDM negatively impacts the blood vessels of the placenta leading to vascular malperfusions which could have several downstream effects such as stillbirth and negative implications for vascular development (Barapatre and Frank, 2025). The wall thickness of GDM placental vessels is larger than that in pregnancies unaffected by diabetes (Jois et al., 2026) which could conceivably be due to changes in SMC phenotype promoting proliferation and migration.

In contrast to this, other studies suggest that gross histological changes in the placenta in response to GDM are limited. The impact of GDM on placental weight has been reported to be higher in a number of studies, however recent examinations have shown no such association (Pooransari et al., 2020; Jois et al., 2026). This suggests that the aforementioned changes such as inflammation and oxidative stress may only be able to be picked up at the cellular level, rather than through histological screening. Similarly, changes to placental vasculogenesis do not have a consensus. Whilst GDM has been shown to alter the paracrine regulation of feto-placental angiogenesis via trophoblast interactions (Loegl et al., 2017), prior studies had suggested that placental angiogenesis was only increased in pre-GDM patients and not those with a GDM diagnosis (Jauniaux and Burton, 2006).

In a bid to address these conflicting findings, multiple recent studies have reported transcriptomic or proteomic datasets on whole placental tissue in the presence and absence of GDM. These have revealed increases in the expression of signalling pathways such as phosphoinositide 3-kinase (PI3K) and Akt (Zhang et al., 2024), and increased expression of insulin-receptor substrate 2 (IRS2 (Colomiere et al., 2009)). GDM placentas present with elevation in many matrix metalloproteinases (MMPs), namely, MMP11 (stromelysin-3), MMP12 (macrophage metalloelastase), MMP14 (activates gelatinases MMP2 and MMP9), and MMP15 which is important for cytotrophoblast invasion (Zhang et al., 2024). It is possible that the altered extracellular matrix tension that this would create could contribute to vascular dysfunction, in particular by altering SMC biomechanics and subsequent phenotype. Finally, the previously mentioned ‘hyperinflammatory’ placenta is defined by increased expression of interleukins, tumour necrosis factor (TNF)-alpha receptors and other markers of inflammation at the tissue level (Radaelli et al., 2003). This hyperinflammation is supported by an increase in the presence of activated B cells in the placenta (Chen et al., 2025) which could cause autoimmune issues during the pregnancy.

These findings indicate complex molecular signalling which influences vascular remodelling. However, information regarding the cell types responsible for these tissue-level changes, is still lacking.

### 6.1 Molecular changes in placental cells

Dysfunction has been identified in different EC subtypes during GDM. In human placental microvascular ECs (hPMECs), microRNA-195-5p contributes to dysfunction by inhibiting vascular endothelial growth factor A (VEGF-A), a key factor in angiogenesis (Zheng et al., 2022). In contrast, increased VEGF expression was observed in placental capillary ECs (Jois et al., 2026). Other features of EC dysfunction in GDM placentas include reduced adenosine transport, lower p44/42 MAPK and Akt phosphorylation, and a switch from insulin receptor IR-A to IR-B expression [reviewed in (Sobrevia et al., 2016)]. Furthermore, overexpression of aquaporin-8 (AQP8) in GDM placentas is involved in vascular structural and functional changes, with high glucose levels inducing EC dysfunction and leading to pathological vascular alterations (Shan et al., 2022).

Whether placental GDM-EC have impaired proliferation or experience higher rates of cell death is unknown, but reduced expression of the endothelial markers platelet endothelial cell adhesion molecule-1 (PECAM-1), von Willebrand factor (vWF) and VE-cadherin has been observed (Byford et al., 2026). Interestingly, GDM has been shown to alter actin organisation of fetoplacental arterial endothelial cells via altered DNA expression and methylation (Cvitic et al., 2018). These findings seem to suggest reduced vascularisation and possible disruption of EC morphology which could lead to dysfunction. Foetal insulin and insulin-like growth factor (IGF)-2 have also been shown to contribute to the upregulation of MMP-14 in placental endothelial cells in GDM, driven by components of the diabetic environment such as hyperglycaemia and altered insulin/IGF signalling (Hiden et al., 2012). Altogether, this highlights the possibility of concurrent inhibitory mechanisms impairing proper vascular development.

The role of endothelial progenitor cells (EPCs) is also crucial in supporting placental vascular health. EPCs are heterogenous precursors to ECs that support vascular homeostasis (Wang X. et al., 2022). In major pregnancy disorders such as GDM, endothelial colony-forming cells (ECFC), a subtype of EPC, migration, proliferation and tube formation are heavily impacted as well as altered p38MAPK signalling and epigenetic changes (Chen et al., 2023).

Similarly to the lack of attention umbilical SMCs have received, comparably little is known about placental SMCs. In humans, maternal spiral arteries undergo a process termed transformation whereby medial SMC are replaced by non-contractile trophoblasts. In GDM, there is evidence of increased trophoblast proliferation (Zhang et al., 2024) with increased VEGF receptor expression (Jois et al., 2026). This indicates cross-talk whereby the altered VEGF EC signalling can impact on SMC (or trophoblast) function. Importantly, there is evidence of increased atherosis/atherosclerosis in spiral arteries of GDM pregnancies (Labarrere et al., 2025), which could be indicative of more widespread pro-atheroslerotic environment within the maternal or foetal circulation. Murine and rodent models have revealed reduced calcium-dependent intermediate potassium (IK_Ca_) channel expression (Gokina et al., 2013; Gokina et al., 2015) in placental GDM-EC, with increased vascular resistance and impaired relaxation (Chirayath et al., 2010; Stanley et al., 2011).

There is emerging evidence suggesting that vascular SMCs are affected via altered differentiation although not linked directly to GDM. Trophoblast cells actively induce SMC phenotypic switching via PDGFRβ, Akt and p44/42 MAPK signalling pathways in rat models (Nandy et al., 2020). While this does not directly address SMC molecular alterations under the hyperglycaemic conditions in GDM, it highlights a mechanistic link between trophoblasts and SMC cell signalling given that GDM can alter trophoblast signalling by regulating GLUT3 translocation in mice models, as well as regulation of feto-placental angiogenesis in humans (Loegl et al., 2017; Xiao et al., 2024).

**FIGURE 3:**
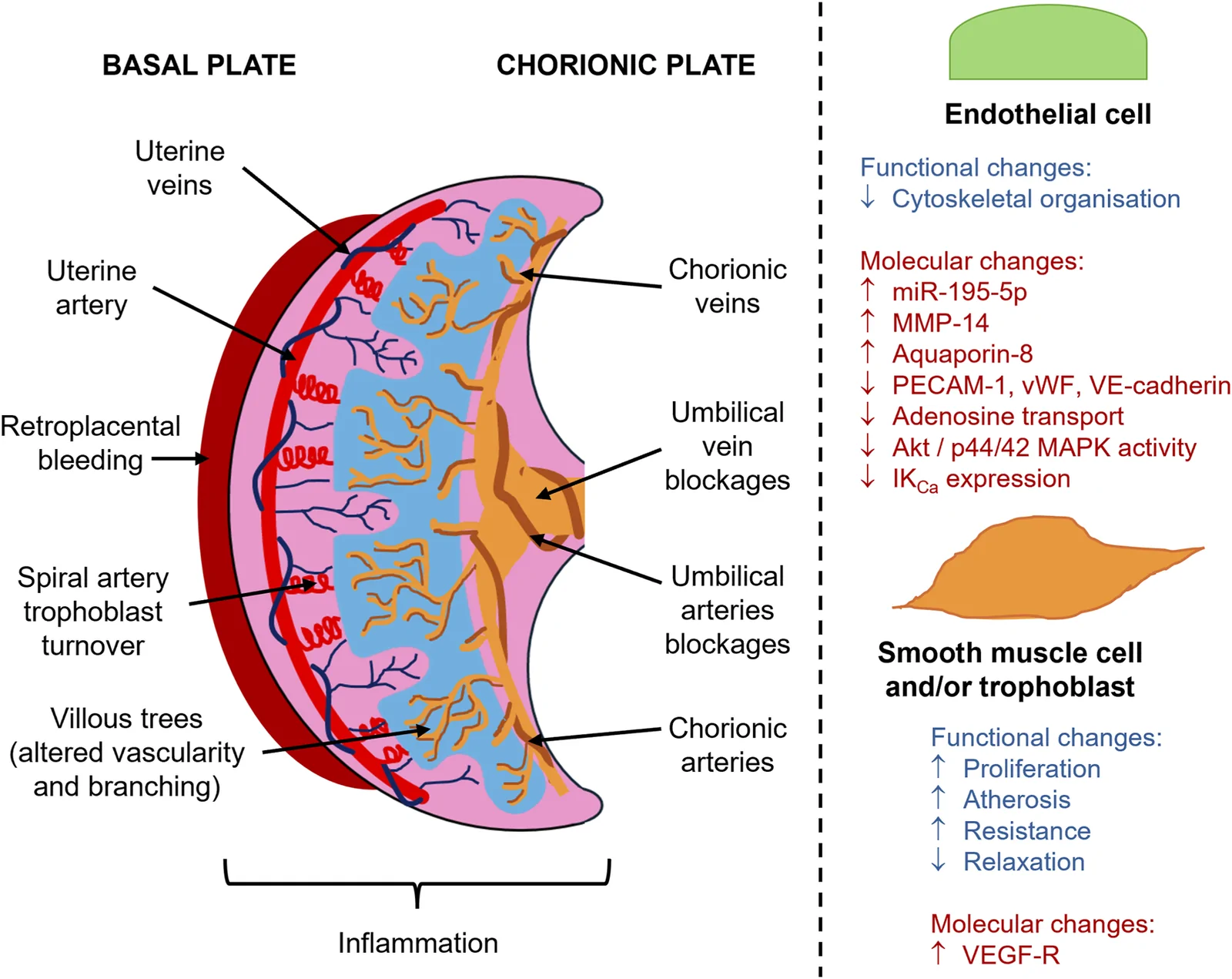
The impact of GDM on the placenta. The GDM placenta tends to be heavier than those seen in non-diabetes pregnancies and is hyperinflammatory, with altered vascularity of the villous trees. Histological changes in the placenta include basal plate vascular turnover and bleeding. At the cellular level, EC have a disorganised cytoskeleton and changes in several signalling pathways. SMC and/or trophoblast biology is also affected by GDM with increased evidence of atherosclerotic processes and vasoconstriction/relaxation. However, these aspects have limited generalisability due to the small number of studies and the lack of specificity over the source of EC and SMC. MAPK, mitogen-activated protein kinase; MMP-14, matrix metalloproteinase-14; PECAM-1, platelet endothelial cell adhesion molecule; VEGF, vascular endothelial growth factor; vWF, von Willebrand factor.

Current knowledge regarding the impact of GDM on placental biology is summarised in Figure 3. One of the main confounding factors that limits our understanding of GDM placentas, other than a lack of widespread study, is the fact that there will be multiple EC and SMC subtypes within the placenta which may have entirely different expression patterns and functional changes. This diversity is not currently captured, yet would greatly enhance our understanding of cell-specific changes.

## 7 Lessons from animal models

Animal models provide mechanistic insights into the impact of GDM on vascular function with studies highlighting how dietary and metabolic alterations in rodents mimic GDM in humans. A high-saturated-fat diet in C57BL/6 has been shown to induce GDM characterised by placental vasculopathy, highlighting the negative impact of maternal metabolic disturbances on the placental vasculature (Bernelot Moens et al., 2012). These vascular alterations may create an adverse intrauterine environment marked by oxidative stress, inflammation, and an impairment in the delivery of oxygen and nutrients. Consistent with this, offspring of Sprague-Dawley rats with streptozotocin-induced (STZ) maternal diabetes exhibit endothelial dysfunction with male STZ offspring showing hypertension, impaired vascular responses driven by arginase-1 dysregulation, and increased oxidative stress. Interestingly, this effect was not seen in female STZ offspring suggesting that the vascular consequences appear to be sexually dimorphic likely as a result of protective mechanisms such as oestrogen-mediated vascular protection (Yu et al., 2021; Zhao et al., 2025). Furthermore, studies on the adult offspring of STZ-induced diabetic Sprague-Dawley rats have shown impaired endothelium-dependent vasodilation and reduced heart rate. Elegant studies involving cross-suckling revealed that these persistent effects were heavily influenced by the dams breast milk (Holemans et al., 1999). Whether this translates into human studies requires further study, as the current evidence suggests that in humans at least, breastfeeding with postnatal diabetes does not have clearcut negative impact on child vascular health (Elbeltagi et al., 2023).

Molecularly, a study on Sprague-Dawley rat models demonstrated that maternal diabetes impairs endothelium dependent relaxation and increases endoplasmic reticulum (ER) and oxidative stress driven by the downregulation of 5'AMP-activated protein kinase (AMPK) and peroxisome proliferator-activated receptor delta (PPAR∂) in offspring. These defects were however shown to be reversible with ER-stress inhibitors or AMPK/PPAR∂ agonists (Luo et al., 2022). While these studies highlight how GDM can induce vascular injury, the vascular biology of animal models differ from humans in key genetic, metabolic and anatomic ways. This is exemplified by the previously mentioned inconsistency regarding breast milk. To better understand the mechanistic link between GDM and maternal/offspring vascular function, studies on human cells will play an important role in deciphering clinically important mechanisms. Two-dimensional monocultures of cells are useful in dissecting individual signalling pathways, but the vascular system is a complex interaction of multiple cell and tissue types. The advent of advanced three-dimensional co-culture models such as vessel-on-a-chip, vascular spheroids or phantoms, where interaction between cell types can be observed, may prove very useful to better understand vascular dysfunction in GDM (Tuna et al., 2026).

## 8 Summary and future perspectives

The evidence that GDM poses a significant risk factor for cardiovascular disease in both mother and child is clear. Despite this, it remains understudied especially in comparison to other metabolic disorders such as T2DM, or other severe pregnancy disorders such as pre-eclampsia. From the research that has been conducted, certain factors appear consistent such as increased inflammation and oxidative stress. However, this alone does not fully explain the persistent impact of GDM on mother and child. Much more research is needed, particularly regarding the cellular and molecular changes that are apparent in GDM.

Adenosine transport is one feature that presents in both EC and SMC, and from both the umbilical cord and the placenta (Sobrevia et al., 2016; Aguayo et al., 2001; Aguayo and Sobrevia, 2000; Salomón et al., 2012). Adenosine has multiple effects on the cardiovascular system including acting as a potent vasodilator and is produced by EC and SMC [reviewed in (Guieu et al., 2020)]. Evidence for increased transport in the umbilical cord, but reduced transport in the placenta suggests a complex tissue-specific picture, whereby simply enhancing or reducing adenosine transport pharmaceutically would not necessarily ameliorate the problem. *In vitro* studies on human placental ECs demonstrated that transport could be improved using insulin (Salomón et al., 2012), but whether this translates to a clinical benefit remains to be seen. Understanding the physiological impact of adenosine transport changes would greatly help in deciding whether or not it could be a relevant therapeutic target.

Another feature that occurs in both umbilical cord and placenta, and their ECs and SMCs, is a reduced expression of potassium channels. In the placenta, K_ATP_ channels are important for regulating vascular tone and so impaired K_ATP_ channels in EC (Gokina et al., 2013; Gokina et al., 2015) could conceivably also contribute to impaired vasorelaxation/vasoconstriction. However, it is important to note that these studies were carried out on animal models and may not be representative of the human placenta. K_ATP_ channels are important for insulin release from pancreatic beta cells and treatments such as potassium channel blockers (e.g., sulfonylureas such as glibenclamide) can be prescribed to women with GDM that is poorly controlled (de Wet and Proks, 2015; Zhang et al., 2019). Given the reduced expression of potassium channels in cord and placental tissue, it is possible that this may be compounded by sulfonylureas and would benefit from further research.

One area that could greatly expand knowledge would be the use of single-cell RNA-Seq or proteomics. As demonstrated above, the placenta is a very complex organ with a rich vascular network. Within the placenta, there will be different vascular ‘niches’ that support the function(s) of the placenta in that specific location. For example, it is highly probable that there are unique expression patterns between chorionic arteries and veins, uterine arteries and veins, and the vessels of the villous trees. While current datasets on bulk RNA-Seq or on non-location-specific ECs have increased our knowledge, this could be better refined using the latest spatial technologies. Multi-omic single cell approaches provide an important opportunity to further elucidate the specific molecular mechanisms and epigenetic modifications underlying metabolic memory. Integrating single-cell RNA-Seq with single-cell ATAC seq for instance will be instrumental in revealing cell-type specific and gene regulation mechanisms by enabling simultaneous characterisation of transcriptional mechanisms and chromatin accessibility in individual cells. This has recently been exemplified in first trimester placentas (Ounadjela et al., 2024) and applying these workflows in different disease states such as GDM will yield a wealth of information.

Finally, we propose that GDM research would benefit from larger histological studies that are sufficiently powered to detect changes with multiple variables such as age, ethnicity and gender of offspring. Whilst the research areas proposed above will allow us to better understand the molecular causes of vascular dysfunction and metabolic memory in GDM, having greater histological understanding could lead to simple tests at point-of-delivery that could be used to introduce early personalised treatments to reduce the impact of metabolic memory and improve cardiovascular and metabolic outcomes for both mother and child.

## Glossary

AIx: augmentation index
AMPK: 5' AMP-activated protein kinase
AQP8: aquaporin-8
ATP: adenosine triphosphate
cAMP: cyclic adenosine monophosphate
cGMP: cyclic guanosine monophosphate
DNA: deoxyribonucleic acid
EC: endothelial cell
ECFC: endothelial colony forming cells
eNOS: endothelial nitric oxide synthase
EPC: endothelial progenitor cell
ER: endoplasmic reticulum
ET-1: endothelin-1
GDM: gestational diabetes
HAPO: hyperglycaemia and adverse pregnancy outcomes
hPMECs: placental microvascular endothelial cells
HUAEC: human umbilical artery endothelial cells
HUASMC: human aortic smooth muscle cells
HUVEC: human umbilical vein endothelial cells
HUVSMC: human umbilical vein smooth muscle cells
IGF: insulin-like growth factor
IR: insulin receptor
LGA: large for gestational age
LV: left ventricular
MAP: mean arterial pressure
MAPK: mitogen-activated protein kinase
MMPs: matrix metalloproteinases
NO: nitric oxide
PECAM-1: platelet endothelial cell adhesion molecule-1
PI3K: phosphoinositide 3-kinase
PPAR∂: peroxisome proliferator-activated receptor delta
PWV: pulse wave velocity
RNA: ribonucleic acid
ROS: reactive oxygen species
SMC: smooth muscle cells
STZ: streptozotocin
T1DM: Type 1 diabetes mellitus
T2DM: type 2 diabetes mellitus
TNF: tumour necrosis factor
VEGF: vascular endothelial growth factor
vWF: von Willebrand factor
